# Can adjunctive corticosteroid therapy improve patient-centered outcomes following third molar surgery? A systematic review

**DOI:** 10.4317/medoral.25177

**Published:** 2022-08-17

**Authors:** Parastoo Parhizkar, Patrick R Schmidlin, Michael M Bornstein, Omid Fakheran

**Affiliations:** 1 Graduate Student, Dental Students’ Research Committee, School of Dentistry, Isfahan University of Medical Sciences, Isfahan, Iran; 2 Clinic of Conservative and Preventive Dentistry, Center of Dental Medicine, University of Zurich, Plattenstrasse 11 8032, Zurich, Switzerland; 3 Department of Oral Health and Medicine, University Center for Dental Medicine Basel UZB, University of Basel, Basel, Switzerland; 4 Department of periodontics, Dental Implants Research center, Dental Research Institute, School of Dentistry, Isfahan University of Medical Sciences, Isfahan, Iran

## Abstract

**Background:**

Third molar surgery is frequently associated with postoperative discomfort such as pain, edema and trismus. We aimed to evaluate the current evidence on the efficacy of adjunctive corticosteroid therapy in improving patient-centered outcomes following third molar surgery.

**Material and Methods:**

This systematic review assessed and searched PubMed, Google scholar, Scopus, web of science, clinicaltrials.gov and Cochrane central for controlled trials, up to May 2021. The primary outcome measures were patient-centered outcomes such as quality of life following the use of adjunctive corticosteroid therapy in third molar removal. Only randomized controlled trials published in English language were included.

**Results:**

A total of 355 studies were initially identified, and 12 studies were finally included. The results showed that both methylprednisolone and dexamethasone decreased postoperative side effects such as pain, trismus, and edema and consequently were improving patient reported outcomes. In this regard, none of the included papers reported any significant statistical difference between these two drugs (*p* > 0.05). The analysis regarding the route of administration for the corticosteroids showed that local and intravenous injection of dexamethasone had equivalent effects, and both methods showed better results as compared to simple oral administration.

**Conclusions:**

Adjunctive use of corticosteroid drugs may improve patient-centered outcomes following third molar surgery. However, there is no significant difference between drugs and routs of administration. Comparing various administration routs, local submucosal injection of dexamethasone seems to be a straightforward, painless and cost-effective adjunctive therapy.

** Key words:**Third molars, corticosteroids, patient-reported outcomes, health related quality of life.

## Introduction

Third molar surgery is one of the most frequently performed interventions in oral surgery. Unfortunately, it is often associated with postoperative complications and morbidity such as facial swelling, pain, trismus, sensitivity, and alveolitis (1,2). This may have a negative impact on both, psychological and biological aspects (3-6). It has been reported that administration of steroidal and non-steroidal anti-inflammatory drugs may subside these common post-operative problems (7). Corticosteroids can minimize the severity of facial swelling, pain, and trismus after surgical removal of impacted third mandibular molars (8-11). Corticosteroids may inhibit production of vasoactive substances and provoke various anti-inflammatory responses including a decrease in the permeability and capillary dilatation (12). There are a number of corticosteroids such as prednisolone (Pred), methylprednisolone (MP) and dexamethasone (DM), which are widely used in oral surgery with various routes of administration and also dosages (13,14).

Many randomized controlled clinical trials (RCTs) and systematic reviews are available, which have reported on the effects of corticosteroid administration on clinician-reported measures and objective outcomes in third molar surgery (15-17). However, there is no consensus in the literature regarding the effect of the adjunctive use of corticosteroids for third molar removal procedures regarding patient-reported outcomes (PROs). PROs provide insights on patients’ experience and perspectives on treatment and outcomes. This can be very useful specifically for patient centered outcomes research (18). PROs are clearly different from clinician-reported and caregiver-reported measures (19). In this context, the Food & Drug Administration (FDA) and the National Quality Forum (NQF) have defined a patient-reported outcome as a direct report from patients, which is neither influenced by clinicians nor anyone else and contains health-related quality of life, functional status, symptoms, and treatment results (20-23).

The primary objective of this article was to systematically review the literature to determine the efficacy of corticosteroids used as adjunctive therapy regarding patient-centered outcomes in third molar surgery. More specifically, the report attempts to answer the following focused question: How effective is adjunctive corticosteroid therapy in prevention of postoperative side effects from the patients' point of view. In this regard, oral health related quality of life (OHRQoL) measures, patient reported discomfort, pain, edema, and trismus were considered as outcomes of interest. Our secondary objective was to summarize the available clinical studies in third molar surgery in which patient-centered outcomes have been assessed. This objective would be of importance to future researchers in terms of what has been tried and what the potentials are for the measuring patients reported outcomes in oral surgery.

## Material and Methods

- Protocol and registration

This systematic review was conducted in accordance with guideline for Preferred Reporting Items for Systematic Reviews and Meta- Analyses (PRISMA) (24). The protocol for the present investigation is registered in the International Prospective Register of Systematic Reviews (PROSPERO; registration code: CRD42020185561).

- PICO question

The PICO question formulated this study was: “In healthy adult patients (> 15 years) who undergo mandibular third molar surgery (P), what is the effect of corticosteroid administration (I) as compared to placebo (C) with regard to patient-reported outcomes (O)?’’

- Eligibility criteria

Only randomized controlled clinical trials published in English were considered without limitation regarding the year of publication. Studies were included if they compared the use of any type of corticosteroid drug to a control group or using a placebo. In addition, studies had to report on patient-reported outcomes without restrictions on how these outcomes were measured. Any other type of publications including letters to the editor, case reports, case-series, retrospective studies, technical reports, conference proceedings, animal or *in vitro* studies, and review papers were excluded. Moreover, studies with insufficient description of patient selection procedure, method of surgery, dosages of corticosteroid and drug administration route were also excluded.

- Search strategy

Two reviewers (P.P and O.F) independently searched the following scientific databases: PubMed, Google scholar, Scopus, web of science, clinicaltrials.gov and Cochrane central register for controlled trials up to May 2021. The search was performed using a combination of medical subject headings (MeSH) and relevant keywords (Table 1). We also manually traced the references included in the literature to obtain additional relevant literature. The first article selection process was based on the reading the titles and abstracts. All the titles and abstracts were independently reviewed by two authors (Kappa coefficient =0.85). Differences in article selection between reviewers (P.P and O.F) were solved by reaching a consensus with the aid of a third reviewer (PS).

- Data extraction

Two authors (P.P and O.F) independently extracted the patient-reported outcomes (OHRQoL, patient reported discomfort, pain, edema, and trismus) and the following data from the papers included: demographic characteristics of study participants (age, gender), study design, follow-up period, publication year, and country of the study. Moreover, the clinical and medical characteristics such as drug therapy protocol (Type of drug, dosage, administration rout, time of administration) and rescue analgesic prescription were also recorded.

**Table 1:**
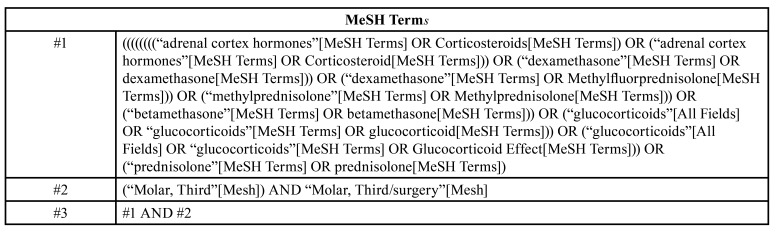
Search terms.

- Risk of bias and quality of the included studies

The risk of bias of the included publications was determined based on the Cochrane Collaboration tool for assessing randomized clinical trials (25). In this regard, all the trials were evaluated for selection bias (random sequence generation and allocation concealment), performance bias (blinding of participants and personnel), detection bias (blinding of outcome evaluation), attrition bias (incomplete outcome data), reporting bias (selective reporting), and other bias (other threat related to origin bias). Finally, the authors’ judgments were categorized as “Low risk” of bias, “High risk” of bias or “Unclear risk” of bias.

- Statistical analysis

Due to substantial heterogeneity in various aspects of included studies, i.e. administration routes, dosages, observation periods, and postoperative measurements, any quantitative post hoc analysis or meta-analysis was not feasible (26). Hence, we decided only to qualitatively describe and summarize the results of included studies.

## Results

- Study selection

**Figure 1:**
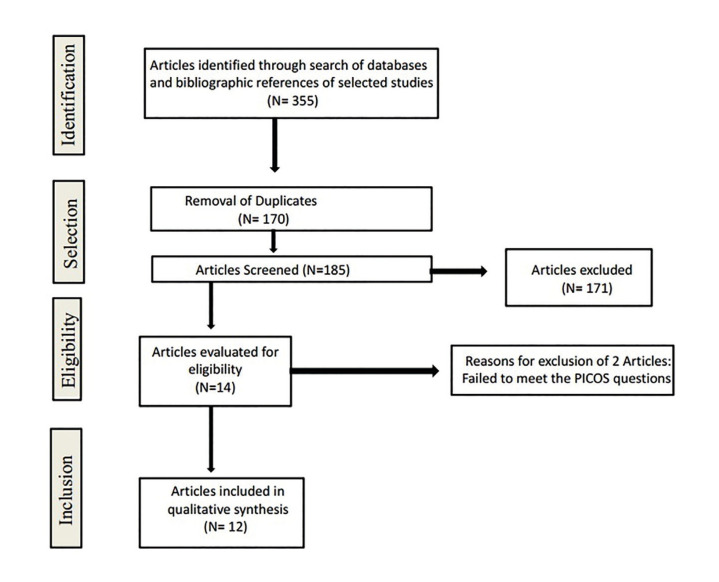
PRISMA flow chart of selection process.

The initial database and hand search yielded a total of 355 entries. No unpublished or ongoing trials were included in this systematic review. After exclusion of duplicates, a total of 185 items were included in title and abstract screening. Afterwards, 14 articles remained to be evaluated for eligibility based on the inclusion criteria. Two articles were excluded from the full-text evaluation because they did not match our PICO question. Thus, the final selection consisted of 12 articles (Fig. 1, Table 2).

**Table 2:**
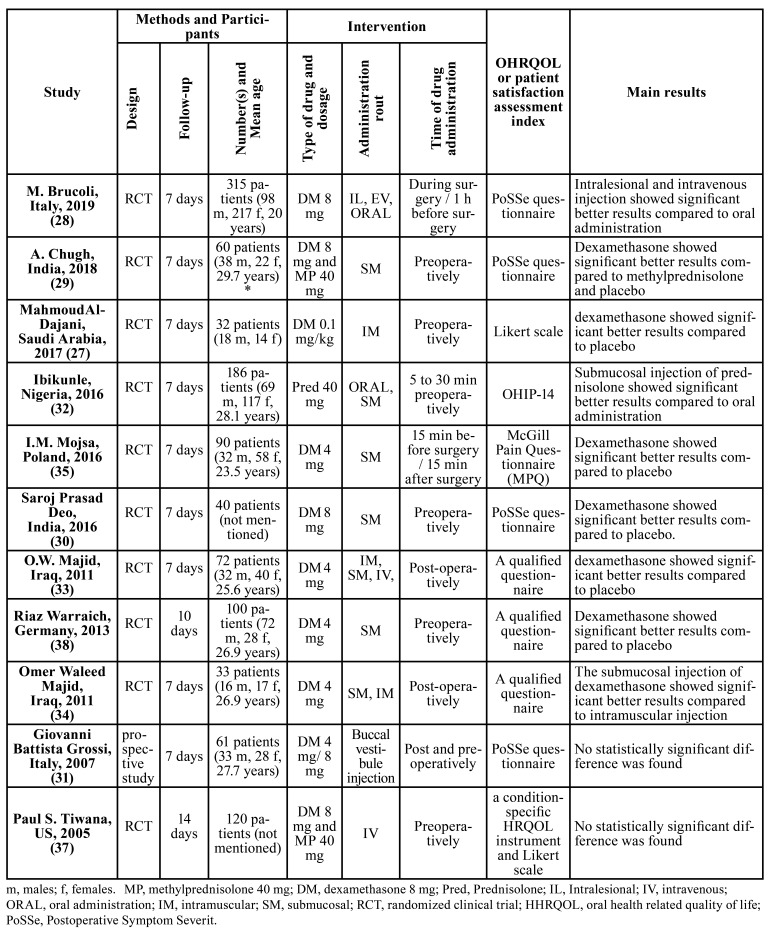
General characteristics and main outcomes of the included studies. (n =12).

- Study characteristics

Of the twelve papers included in this systematic review (Table 2) (27-38), two studies compared the patient-centered outcomes related to third molar surgery following administration of various types of corticosteroids (29,37). Five studies assessed different administration routes (28,32-34,36). One paper assessed the impact of different dosages of a single corticosteroid drug (DM) (31). Various types of patient-reported outcomes such as severity of pain, edema, trismus, level of patient satisfaction and OHRQoL have been employed in the included studies (Table 2). Among all, only five studies considered quality of life as the patient-reported measure (29,30,32,34,38). The number of patients included in each study varied between 32 and 350. The follow-up duration was at least six days up to fourteen days in the studies.

- Quality assessment

Three of the studies had good methodological design with low risk of bias (Table 3) (27,28,38). However, nine studies showed a fair methodological quality (29-37). In the studies with fair methodological quality, possible areas of bias were mainly related to ‘sequence generation’, ‘allocation concealment’ and, ‘blinding of participants and outcome assessors’.

**Table 3:**
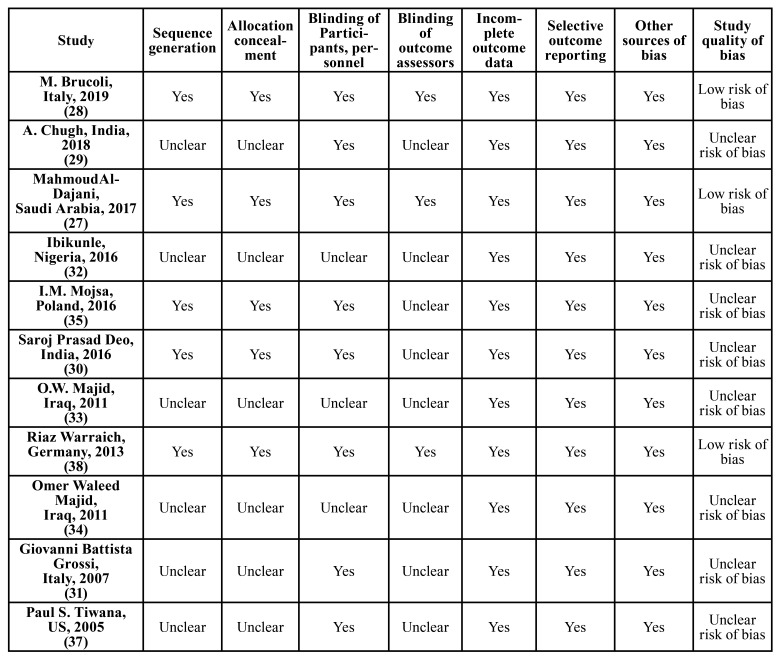
Findings from the risk of bias assessment using Cochrane Collaboration tool for assessing randomized clinical trials.

- Outcome measure tools

All included studies used validated assessment tools. The Postoperative Symptom Severity (PoSSe) scale was used in 4 articles (28-31). Likert scale was also filled by patients in 2 studies (27,37). McGill Pain Questionnaire (MPQ) (35) and OHIP-14 (32) were the other questionnaires used. Five out of the twelve studies did not specify measures used in this regard (33,34,36-38).

- Types of corticosteroids

Most studies in this review used DM with various doses for managing postoperative discomforts after third molar surgery. However, in two studies, the results of MP and Pred prescriptions were compared.

Two studies compared the impact of different types of corticosteroids on patient reported outcomes (37,29). The results of these studies were largely similar for most of the measures. One of these studies evaluated the differences in eating, speech, sensation, appearance, sickness, and interference with daily activities between placebo, DM 8 mg (Submucosal injection), and MP 40 mg (Submucosal injection) groups(29). Considering the limitation of this RCT, the authors suggested that the submucosal injection of 8 mg dexamethasone is an effective therapeutic strategy to reduce swelling and pain after the surgical removal of impacted lower third molars (29). In the other study, submucosal injection of DM sodium phosphate 8 mg and MP sodium succinate 40 mg as interventional groups were compared with control group. The results of this study showed that no statistically significant differences were found between groups. However, both types of drugs could reduce patient reported problems comparing to placebo group (Table 2) (33). In addition, both of the studies confirmed the positive effect of corticosteroids in comparison to placebo with regard to patient satisfaction.

- Different administration routes

The selected papers in this review used various routs of administration for corticosteroids including submucosal injection, local parenteral route (injection in the masseter muscle), intravenous injection, oral administration and endoalveolar injection. In this regard, one of the studies showed that the intravenous group had the least impairment reported by patients and the best patient centered outcomes followed by oral and submucosal groups in an ascending order (*P* < 0.01) (33)

A significant improvement of oral health-related Quality of Life (OHRQoL) was found in either oral administration or submucosal injection of Pred groups. However, submucosal injection of Pred showed significantly better results in (OHRQoL) improvement than oral administration of Pred (*P* = 0.001) (32). Based on the results obtained from the PoSSe questionnaire in another study, local parenteral route and intravenous injection of DM showed significantly better results than oral administration of this drug (*P* < 0.05) (28). Regarding the different locations of local injection, Shirani *et al*. assessed the effectiveness of DM injection into the medial pterygoid or gluteal muscles with the aim of preventing postoperative complications after third molar surgery. The results of this investigation showed that both routs of administration of the drug were significantly effective with regard to reducing postoperative pain, swelling, and changes in appearance comparing to the control group (36).

In one of the included studies in this review assessing the intramuscular route of injection for DM, a single-dose (0.1 mg/kg) was prescribed to assess fourteen different patient-centered outcomes such as patients' discomfort, limitation of oral function, and limitation of daily activities(27). Less difficulty in eating (P ≤ .024), less difficulty in enjoying food (P ≤ .005), less difficulty in speech (P = .043), less absence from school or work (P ≤ .016), and less disruption of daily activity (P ≤ .042) were detected as a result of a single-dose intramuscular injection of DM compared to placebo. It should be mentioned that the results of this study showed no significant difference in sleep disturbance (27). In another study, the outcomes of intramuscular and submucosal (buccal mucosal region of the third molar) injections of 4 mg DM administered immediately after surgery were compared. Based on the results of this clinical trial, the patient reported outcomes including swelling, pain, and QOL measures in the immediate postoperative period were significantly improved in both of the test groups compared to the controls. Interestingly, only the submucosal group showed significant improvement of trismus compared to the controls (34).

- Different dosages of submucosal administration

Regarding the comparison of various doses of DM submucosal administration (4 mg and 8 mg), the patients’ perception of the severity of symptoms was assessed by PoSSe scale in one of the included studies. Among the seven domains of this instrument, just facial edema criteria showed a statistically significant reduction for both dexamethasone groups compared with to the control group. No significant differences were observed between the 2 different dosage regimens of dexamethasone (31). In another study, the effectiveness of 4 mg submucosal injection of DM on patient quality of life was assessed by Warraich *et al*. Based on the results of this study, the quality of life of patients in DM group was significantly higher in comparison with patients in the control group following the surgery (38).

Comparing the effectiveness of preoperative and postoperative submucosal injection of 1 ml DM (4 mg/ml), postoperative pain was evaluated using the McGill Pain Questionnaire. The results of this study revealed a significant better pain control in postoperative DM than in preoperative DM group (35).

In another study, quality of life was significantly improved by submucosal injection of a single dose of 8 mg DM compared to the placebo group. However, only three (Eating, Appearance and Sickness subscale) out of seven subscales revealed statistically significant difference between these two groups (30).

## Discussion

The present systematic review evaluated the effects of corticosteroids on patient-reported outcomes in mandibular third molar surgery. A total of twelve RCTs were included in the final set of selected articles (27-38). Firstly, the data collected for this review showed that a large variety of scales and measurement tools were used to assess Patient-Reported Outcomes (PROs). One of them was the Post-Operative Symptom Severity (PoSSe) scale that represents an exclusive measure for evaluating the oral health-related quality of life following third molar surgery. This questionnaire contains various queries regarding subjective factors that may affect the patients’ quality of life (40). The PoSSe scale is a valid, reliable and responsive index for surgical outcomes and their impact on the quality of life from the patients’ perspective (39,40). This measurement was employed in four of the included studies (28-31).

Another measurement tool that was utilized in two studies was the Likert scale, which simply asks to what extent people agree with or accept an observation using a 5- or 7-point scale. Scale 1 represents a strong agreement, while scales 5 or 7 express strong disagreement, and 3 is a manifestation of being neutral (41). The third measurement tool applied was the Oral Health Impact Profile (OHIP-14), one of the most popular Patient-Reported Outcome Measures (PROMs) in dental medicine, which was used in only one of the included studies (32). OHIP-14 measures individuals’ perceptions regarding the effect of oral condition on their quality of life and consists of 14 items organized into seven subscales (42).

Based on literature, Pred, MP and DM are broadly used to diminish postoperative side effects, such as pain, trismus, and edema. Both medications could improve patients’ satisfaction, but there was no significant difference between these two types of corticosteroids with regard to patient-centered outcomes (29,37).

Several studies have assessed various administration routes as the main variable, but their results were inconsistent. This can be explained by variations in methods and instruments that have been used for assessing patient-reported outcomes. However, it has been demonstrated unanimously that oral Tablets are not as effective as submucosal injection, injection in the masseter muscle, and intravenous injections (28). In confirmation of this, the authors of a recent systematic review reached a similar conclusion. They concluded that a single pre-operative dose of oral corticosteroids would not be clinically effective in reducing pain, trismus and edema following lower third molar surgical extraction(43). In the study of Majid *et al*., five different routes of administration were compared, and all treatment groups revealed statistically significant differences in all subscales of the QOL questionnaire compared to the control group. Among these, intravenous administration showed significantly better results in comparison to the other ones (33). Nevertheless, local submucosal injection of DM in the buccal mucosal region of third molar is quite simple, safe, less invasive, painless, cost-effective and efficient. Finally, dentists are recommended to employ only one route based on their competence and expertise (33).

Despite the fact that all selected studies in this review were randomized clinical trials, they disclosed a great heterogeneity in terms of sample size, demographic criteria, outcome measures and evaluation tools. Thus, the results of this systematic review should be cautiously interpreted. This was also the reason, why no meta-analysis could be performed. Further well-designed randomized clinical trials containing comparable protocols, larger sample sizes, more generalizable and adequate patient-reported outcome measures are needed also to more reliably determine the optimal dosage of corticosteroids and the administration route improving the immediate quality of life after surgical removal of third molars.

Some limitations were observed in the present systematic review. We have included just the articles published in English language in our review. Various numbers of removed teeth per patient was reported among the included studies in this review. The impact of surgery on patient centered outcomes may have been different for those submitted to both side mandibular third molar surgeries compared to those who had only one tooth removed. Furthermore, variations in the included studies regarding the degree of difficulty of surgery were not assessed here, which may influence possible complications and side effects.

## Conclusions

This review showed that all types of administered corticosteroids result in an improved patient satisfaction, but there were no significant differences for the DM, Pred, and MP groups. In comparison to other administration routes, the submucosal injection of DM in the buccal mucosal region of third molar is a straightforward, painless, and cost-effective method.
